# Prevalence and modifiable risk factors of cognitive frailty in patients with chronic heart failure in China: a cross-sectional study

**DOI:** 10.1186/s12872-024-03753-x

**Published:** 2024-02-07

**Authors:** Jiayi Xu, Luwei Xiang, Huichao Zhang, Xing Sun, Dongmei Xu, Die Wu, Chen Chen, Yixiong Zhang, Zejuan Gu

**Affiliations:** 1 School of Nursing, Nanjing University of Chinese Medicine, 138 Xianlin Road, Nanjing, Jiangsu 210023 China; 2 The Second Hospital of Nanjing, Affiliated to Nanjing University of Chinese Medicine, 1-1 Zhongfu Road, Nanjing, Jiangsu 210003 China; 3 Nanjing Women and Children’s Healthcare Hospital, 123 Tianfei Road, Nanjing, Jiangsu 210004 China; 4 Department of Cardiology, The First Affiliated Hospital of Nanjing Medical University, 300 Guangzhou Road, Nanjing, Jiangsu 210029 China; 5 Jiangsu Province Hospital of Chinese Medicine, 155 Hanzhong Road, Nanjing, Jiangsu 210029 China; 6 The First Clinical Medical College, Nanjing University of Chinese Medicine, 282 Hanzhong Road, Nanjing, Jiangsu 210029 China; 7 School of Nursing, Nanjing Medical University, 101 Longmian Road, Nanjing, Jiangsu 211166 China; 8 Department of Nursing, The First Affiliated Hospital of Nanjing Medical University, Jiangsu Province Hospital, 300 Guangzhou Road, Nanjing, Jiangsu 210029 China

**Keywords:** Chronic heart failure, Cognitive impairment, Frailty, Prevalence, Risk factors

## Abstract

**Background:**

Cognitive frailty (CF) is currently a significant issue, and most of the associated factors discovered in current studies are not modifiable. Therefore, it is crucial to identify modifiable risk factors that can be targeted for interventions in patients with chronic heart failure (CHF). This study aimed to investigate the prevalence and modifiable risk factors of CF in CHF patients in China.

**Methods:**

In this cross-sectional study, we sequentially enrolled patients diagnosed with CHF. CF served as the dependent variable, assessed through the Montreal Cognitive Assessment (MoCA) Scale and the FRAIL Scale. The independent variable questionnaire encompassed various components, including general demographic information, the Social Support Rating Scale (SSRS), the Simplified Nutrition Appetite Questionnaire (SNAQ), the Hamilton Depression Scale (HAMD), the Hamilton Anxiety Scale (HAMA), and the Minnesota Living with Heart Failure Questionnaire (MLHFQ). Logistic regression analysis was employed to identify independent factors contributing to CF.

**Results:**

A total of 271 patients with CHF were included in the study. The overall prevalence of CF was found to be 49.4%, with 28.8% of patients exhibiting potentially reversible cognitive frailty and 20.7% showing reversible cognitive frailty. Among middle-young CHF patients, 10.7% had reversible cognitive frailty and 6.4% had potentially reversible cognitive frailty, with a prevalence of CF at 17.1%. Logistic regression analysis revealed that body mass index (*OR* = 0.826, 95%CI = 0.726–0.938), blood pressure level (*OR* = 2.323, 95%CI = 1.105–4.882), nutrition status (*OR* = 0.820, 95%CI = 0.671–0.979), and social support (*OR* = 0.745, 95%CI = 0.659–0.842) were independent factors associated with CF (*p* < 0.05).

**Conclusions:**

We observed a relatively high prevalence of CF among Chinese patients diagnosed with CHF. Many factors including BMI, blood pressure level, nutrition status, and social support emerging as modifiable risk factors associated with CF. We propose conducting clinical trials to assess the impact of modifying these risk factors. The outcomes of this study offer valuable insights for healthcare professionals, guiding them in implementing effective measures to improve the CF status in CHF patients during clinical practice.

## Introduction

Chronic heart failure (CHF) is a persistent state of heart failure and a major cause of death from various cardiovascular diseases. According to the European Heart Association’s Guidelines for Diagnosis and Treatment of Acute and Chronic Heart Failure, the prevalence of heart failure in developed countries ranges from 1.5 to 2.0% [1]. According to the Report on Cardiovascular Health and Diseases in China 2021: an Updated Summary, there were 8.9 million patients with heart failure in China [2]. Heart failure is a chronic and recurring disease, with a high 30-day readmission rate of up to 20–25% and a five-year survival rate of 56.7% [3, 4]. The development of CHF is a lengthy process with a poor overall prognosis.

The concept of frailty is increasingly being considered in the study and treatment of CHF patients. Frailty is an age-related clinical syndrome characterized by reduced physiological reserves in stressful situations, constituting a state of vulnerability that involves a higher risk of adverse events [5]. It adversely impacts the mortality of heart failure patients [6], seriously interferes with the recovery process and increases the risk of suicide in CHF patient [7]. Frailty encompasses both physical and mental aspects, and research indicates a close correlation between physical frailty in CHF patients and cognitive impairment [8]. While physical frailty and cognitive impairment were previously studied separately, recent research has highlighted their close relationship [9].

Thus, the concept of cognitive frailty (CF) was proposed to combine physical frailty and cognitive impairment. CF is characterized by the coexistence of physical frailty and cognitive impairment, with a Clinical Dementia Rating (CDR) score of 0.5, but excluding Alzheimer’s disease or other forms of dementia [10]. Previous research has shown that CF is prevalent in patients with cardiovascular disease and can have a negative impact on their clinical outcome, functional status and quality of life [11]. A study of Japanese heart failure patients over the age of 65 found that those with CF had a 1.55 times higher risk of poor prognostic outcomes in the first year compared to those without CF [12]. Previous studies indicated that CF could be reversible [13], thus, identifying and responding to risk factors for CF can help to slow down or even reverse its progression. However, the associated factors discovered in current studies of CF, such as age, education level, etc., are not intervenable [14]. A review shows that social status, nutrition status, physical and cognitive activities and functional status are factors shown to be associated with CF [15]. However, the effect of depression in CF remains controversial and we need to explore future [16, 17]. Social support has been shown to be an influential factor for CF in hypertensive patients [18] and other studies have found the effects of BMI and nutrition status of CF [19]. This study includes some known influencing factors of CF and hypothesizes that these factors are also relevant for patients with CHF. Therefore, this study aims to investigate the prevalence and modifiable risk factors of CF in patients with CHF in China. We hope to draw the attention of medical staff and improve the prognosis of CHF patients by intervening on modifiable risk factors of CF and to improve the recovery process of patients and reduce mortality.

## Methods

### Design and participates

This cross-sectional was conducted in the Cardiology Department of Jiangsu Province Hospital from August 2022 to February 2023. Patients with CHF were enrolled sequentially using a convenience sampling method. The inclusion criteria were as follows: (a) meeting the diagnostic criteria of CHF in Chinese guidelines for the diagnosis and treatment of heart failure 2018 [20]; (b) being ≥ 18 years old; (c) having a New York Heart Association functional classification of I ~ IV; (d) being able to communicate normally; (e) volunteering and signing an informed consent form. The exclusion criteria were as follows: (a) having dementia or other mental illnesses; (b) having a medical history of severe liver, kidney, brain etc. or other physical diseases; (c) being unable to cooperate with this study. The research protocol was approved by the Ethics Committee of the First Affiliated Hospital of Nanjing Medical University (2022-SR-502).

The questionnaire was collected without hindering the treatment and rest of patients. During data collection, the investigators performed face-to-face conversation to give corresponding explanations in time to ensure the integrity of data. To facilitate communication and future follow-up studies, each patient was individually marked during the survey. Subsequent to data collection, the collectors promptly organized and verified the data. All information underwent a thorough recheck and was independently entered into the computer by two investigators.

### General demographic data

We utilized a self-made questionnaire to obtain the sociodemographic data from patients, including age, gender, education level, marital status, work status, smoking and drinking habits, body mass index (BMI, kg/m^2^), and more. Clinical information was obtained from electronic medical records, including the New York Heart Association (NYHA) functional classification, CHF course, physician-diagnosed comorbidities (such as hypertension, diabetes, coronary artery disease, etc.), and left ventricular ejection fraction (LVEF) (%), etc. For some continuous variables, we applied a transformation. We found that the course of disease was measured by a classification [18], which has not been applied in the study of CHF, although it has been done clinically. In line with the education system in China, we categorized education levels into five groups. According to guidelines [20], we used a cut-off point of 125 pg/ml to group NT-proBNP. LVEF was divided into three categories: heart failure with reduced ejection fraction (HFrEF, LVEF < 40%), heart failure with mid-range ejection fraction (HFmrEF, LVEF between 40% and 49%) and heart failure with preserved ejection fraction (HFpEF, LVEF ≥ 50%).

### Cognitive frailty

CF refers to the combination of physical frailty (PF) and cognitive impairment, excluding dementia. To assess cognitive function, we utilized the Montreal Cognitive Assessment (MoCA) (Beijing version) [21], which consists of 30 points and a score of less than 26 indicates cognitive impairment. The MoCA evaluates cognitive abilities in various areas, including spatial/executive thinking, naming, memory, attention, language, abstraction, delayed recall and orientation. The Cronbach’s α coefficient for this scale is 0.818. Measurement of the frailty using the FRAIL Scale [22] proposed by the experts of the International Nutrition, Health and Work Group in 2008. It is based on the frailty phenotype and Frailty Index, and consists of 36 items selected from the Short Form of medical outcome study, as well as some items on disease and weight loss. The resulting 5-item scale assigns 1 point to each item, with a total score ranging from 0 to 5. A score of 3 or higher indicates frailty, 1–2 indicates pre-frailty, and 0 indicates no frailty. The Cronbach’s α coefficient for this scale is 0.826. According to Ruan’s definition of CF, this study distinguishes between reversible cognitive frailty and potentially reversible cognitive frailty [23]. Patients who were in physical frailty or pre-frailty and had a MoCA score of less than 26 were classified as potentially reversible cognitive frailty. For those with frailty or pre-frailty who had a normal MoCA score, subjective cognitive frailty was considered reversible if they answered “Yes” to the question “Do you think you have memory decline compared to a year ago?”. Patients who responded with a “No” to the question or those who exhibited no signs of frailty or cognitive impairment were categorized as none cognitive frailty. All of our investigators have received training in this area.

### Social support

The Social Support Rating Scale (SSRS), developed by Xiao in 1986, was utilized to evaluate social support [24]. The scale comprises 10 items and three dimensions: objective support (three items), subjective support (four items) and support utilization (three items). The objective support score ranges from 4 to 16, the subjective support score ranges from 5 to 38, and the support utilization score ranges from 3 to 12. A higher total score indicates a higher level of support. The SSRS scale demonstrates good reliability and validity, with a Cronbach’s α coefficient of 0.941. This scale was employed to gain a better understanding of patients’ social support and its correlation with mental health, mental illness, and various physical conditions.

### Nutrition

The Simplified Nutrition Appetite Questionnaire (SNAQ) was developed from the Appetite Scale (CNAQ) by the Community Committee for Long-term Nutrition Care Strategy [25]. It consists of only four items, each with five response options represented by letters A to E. The scoring method used is a Likert five-level scale, with scores ranging from 4 to 20 points. A lower score indicates a poorer appetite and an increased risk of weight loss. A score of ≤ 14 suggests a higher risk of malnutrition, with more than a 5% weight loss in the last six months. The Cronbach’s α coefficient for this scale is 0.72.

### Anxiety and depression

The Hamilton Depression Scale (HAMD) [26] and the Hamilton Anxiety Scale (HAMA) [27] were utilized to evaluate depression and anxiety. The HAMD scale comprises seven factor structures, including anxiety/somatization, body mass, cognitive impairment, day-night change, delay, sleep disturbance and sense of hopelessness. Scores between 8 and 20 indicate mild depression, 21 to 35 indicate moderate depression, and scores greater than 35 indicate severe depression. A score of 8 or below on the HAMD is considered clinical remission, with a Cronbach’s α coefficient of 0.92. The HAMA scale consists of two component structures: psychological anxiety and physical anxiety. Trained researchers score each item on a 5-point scale. A score of 14 to 21 indicates mild anxiety, 22 to 29 indicates moderate anxiety, and scores greater than 29 indicate severe anxiety.

### Health-related quality of life

The term “health-related quality of life” refers to a patient’s personal experience and feelings regarding heart failure and its treatment’s impact on their daily life. The Minnesota Living with Heart Failure Questionnaire (MLHFQ) was utilized to assess it [28]. This 21-item questionnaire is specifically designed to evaluate the physical, socioeconomic, and psychological impairment perceived by patients with HF. Each item has six response options, ranging from 0 to 5, with higher scores indicating poorer HRQOL. The scale is the most commonly used tool for measuring HRQOL in heart failure studies, and it has demonstrated acceptable reliability and validity, with a Cronbach’s α coefficient of 0.91.

### Data analysis

General demographic data, cognitive frailty, nutrition, anxiety, depression and health-related quality of life of patients were analyzed descriptively. Normally distributed data were expressed as mean ± standard deviation, while non-normally distributed data were expressed as percentage or median (25th and 75th percentiles). Categorical variables were presented as frequency and percentage. Pearson correlation analysis was employed for continuous variables with a normal distribution, while Spearman correlation analysis was used for continuous variables with a non-normal distribution. Following the correlation analysis, independent variables with a *p*-value < 0.05 were included in logistic regression analysis to identify the independent factors associated with cognitive frailty. Data analysis was conducted using SPSS 25.0.

## Results

### Descriptive statistics

A total of 271 patients were included in this study, of which 78 patients exhibited potentially reversible cognitive frailty, resulting in a prevalence of 28.8%. Among these patients, 56 were diagnosed with reversible cognitive frailty, resulting in a prevalence of 20.7%. The overall prevalence of CF in CHF patients was found to be 49.4%. In middle-young CHF patients, 10.7% exhibited reversible cognitive frailty, while 6.4% displayed potentially reversible cognitive frailty. The total prevalence of cognitive frailty in this group was 17.1%.

### Differences in physiological functioning and psychological characteristics of CHF patients with different degrees of CF

Table 1 presents additional general data and disease-related information. Univariate analysis revealed that age, caregiver status, education level, marital status, combined with hypertension and cerebral disease, course of CHF, newly diagnosed CHF, blood type, LVEF, NYHA functional classification, Barthel self-care ability, BMI, nutrition status, and degree of anxiety and depression degree were all significantly associated with CF (*p* < 0.05).

**Table 1:** General data and univariate analysis of patients with CHF

Variable	Number	Non-CF (n, %)	R-CF (n, %)	PR-CF (n, %)	X^2^/H	*p*-Values
Sex						
Male	165(60.9)	90(65.7)	33(58.9)	42(54.3)	3.043*	0.218
Female	106(39.1)	47(34.3)	23(41.1)	36(45.7)		
Age group						
18–59	64(23.6)	53(38.7)	6(10.7)	5(6.4)	59.733**	**< 0.001**
60–69	68(25.1)	36(26.3)	18(32.1)	14(17.9)		
70–79	100(36.9)	45(32.8)	27(48.2)	28(35.9)		
≥ 80	39(14.4)	3(2.2)	5(8.9)	31(39.7)		
Caregiver						
Spouse	125(46.1)	73(53.3)	30(53.6)	22(28.2)	7.572**	**0.023**
Children	103(38.0)	39(28.5)	19(33.9)	45(57.7)		
Parent	21(7.7)	12(8.8)	3(5.4)	6(7.7)		
Relative	7(2.6)	3(2.2)	1(1.8)	3(3.8)		
Others	15(5.5)	10(7.3)	3(5.4)	2(2.6)		
Education level						
Illiteracy	22(8.1)	9(6.6)	2(3.6)	11(14.1)	7.379**	**0.025**
Primary	60(22.1)	24(17.5)	14(25.0)	22(28.2)		
Middle	157(57.9)	83(60.6)	38(67.9)	36(46.2)		
College	32(11.8)	21(15.3)	2(3.6)	9(11.5)		
Graduate	1(0.4)	1(0.7)	0(0.0)	0(0.0)		
Marital status						
Unmarried	7(2.6)	6(4.4)	0(0.0)	1(1.3)	7.649**	**0.022**
Married	239(88.2)	121(88.3)	54(96.4)	64(82.1)		
Divorced	2(0.7)	2(1.5)	0(0.0)	0(0.0)		
Widowed	23(8.5)	8(5.8)	2(3.6)	13(16.7)		
Combined with hypertension						
Yes	136(50.2)	59(43.1)	29(51.8)	48(61.5)	6.857*	**0.032**
No	135(49.8)	78(56.9)	27(48.2)	30(38.5)		
Combined with diabetes						
Yes	71(26.2)	31(22.6)	17(69.6)	23(29.5)	1.841*	0.398
No	200(73.8)	106(77.4)	39(30.4)	55(70.5)		
Combined with Coronary Heart Disease						
Yes	94(34.7)	43(31.4)	20(35.7)	31(39.7)	1.565*	0.457
No	177(65.3)	94(68.6)	36(64.3)	47(60.3)		
Combined with cerebral disease						
Yes	51(18.8)	20(14.6)	7(12.5)	24(30.8)	10.352*	**0.006**
No	220(81.2)	117(85.4)	49(87.5)	54(69.2)		
Course of CHF						
≤ 1year	145(53.5)	84(61.3)	24(42.9)	37(474.)	9.050**	**0.011**
1.1-5 year	64(23.6)	31(22.6)	11(19.6)	22(28.2)		
5.1–10 year	43(15.9)	14(10.2)	15(26.8)	14(17.9)		
10.1–15 year	17(6.3)	6(4.4)	6(10.7)	5(6.4)		
>15year	2(0.7)	2(1.5)	0(0.0)	0(0.0)		
Newly diagnosed CHF						
Yes	146(53.9)	84(61.3)	23(41.1)	39(50.0)	7.216*	**0.027**
No	125(46.1)	53(38.7)	33(58.9)	39(50.0)		
NT-proBNP						
0-125	20(7.4)	10(7.5)	5(8.9)	5(6.2)	0.305*	0.859
>125	251(92.6)	124(92.5)	51(91.1)	76(93.8)		
LVEF						
HFrEF	90(33.2)	56(40.9)	15(26.8)	19(24.4)	10.987**	**0.004**
HFmrEF	68(25.1)	36(26.3)	16(28.6)	16(20.5)		
HFpEF	113(41.7)	45(32.8)	25(44.6)	43(55.1)		
Blood type						
A	78(28.8)	34(24.8)	23(41.4)	21(26.9)	7.962**	**0.019**
B	81(29.9)	39(28.5)	17(30.4)	25(32.1)		
AB	50(18.5)	28(20.4)	10(17.9)	12(15.4)		
O	62(22.9)	36(26.3)	6(10.7)	20(25.6)		
NYHA functional classification						
I	6(2.2)	4(2.9)	2(3.6)	0(0.0)	9.620**	**0.008**
II	150(55.4)	79(57.7)	35(62.5)	36(46.2)		
III	94(34.7)	47(34.3)	17(30.4)	30(38.5)		
IV	21(7.7)	7(5.1)	2(3.6)	12(15.4)		
Barthel self-care ability						
No	21(7.7)	17(12.4)	3(5.4)	1(1.3)	23.710**	**< 0.001**
Low level	72(26.6)	44(32.1)	16(28.6)	12(15.4)		
Medium level	134(49.4)	60(43.8)	32(57.1)	42(53.8)		
High level	44(16.2)	16(11.7)	5(8.9)	23(29.5)		
BMI						
< 18.5 kg/m^2^	9(3.3)	2(1.5)	2(3.6)	5(6.4)	6.110**	**0.047**
18.5–23.9 kg/m^2^	153(56.5)	66(48.2)	34(60.7)	53(67.9)		
≥ 24 kg/m^2^	109(40.2)	69(50.4)	20(35.7)	20(25.6)		
Smoking						
Yes	32(11.8)	19(13.9)	7(12.5)	6(7.7)	5.112**	0.078
No	181(66.8)	84(61.3)	37(66.1)	60(76.9)		
Quit	58(21.4)	34(24.8)	12(21.4)	12(15.4)		
Drinking alcohol						
Yes	18(6.6)	12(8.8)	4(7.1)	2(2.6)	1.697**	0.428
No	220(81.2)	107(78.1)	46(82.1)	67(85.9)		
Quit	33(12.2)	18(13.1)	6(10.7)	9(11.5)		
Malnutrition risk						
No	153(56.6)	89(65.0)	34(60.7)	30(38.5)	14.721*	**0.001**
Yes	118(43.5)	48(35.0)	22(39.3)	48(61.5)		
Depression						
No	87(32.1)	63(46.0)	12(21.4)	12(15.4)	27.675**	**< 0.001**
Low level	176(64.9)	73(53.3)	42(75.0)	61(78.2)		
Medium level	8(3.0)	1(0.7)	2(3.6)	5(6.4)		
Anxiety						
No	202(74.5)	114(83.2)	41(73.2)	47(60.3)	14.893**	**0.001**
Low level	65(24.0)	23(16.8)	15(26.8)	27(34.6)		
Medium level	4(1.5)	0(0.0)	0(0.0)	4(5.1)		

Note: *presents chi-square test; ** presents Kruskal-Wallis Test

Abbreviations: Non-CF = none cognitive frailty; R-CF = reversible cognitive frailty; PR-CF = potentially reversible cognitive frailty; CHF = chronic heart failure; LVEF = left ventricular ejection fraction; HFrEF = heart failure with reduced ejection fraction; HFmrEF = heart failure with mid-range ejection fraction; HFpEF = heart failure with preserved ejection fraction; NYHA = New York Heart Association; BMI = body mass index

### Correlations of physiological function and psychological characteristics with CF in CHF patients

The study found that the average SSRS score of CHF patients was 34.92 ± 3.72, while the average MLHFQ score was 50.99 ± 12.83. Correlation analyses revealed that CF was positively associated with age (*r* = 0.551, *p* < 0.001), anxiety (*r* = 0.337, *p* < 0.001), depression (*r* = 0.364, *p* < 0.001), and health-related quality of life (*r* = 0.272, *p* < 0.001). Conversely, CF demonstrated negative associations with BMI (*r* = -0.220, *p* < 0.001), nutrition state (*r* = -0.252, *p* < 0.001), and social support (*r* = -0.503, *p* < 0.001). The results are presented in Table 2.

**Table 2:** Correlations of physiological function and psychological characteristics with CF in CHF patients

Characteristic	FRAIL Scale score		MoCA score		Cognitive frailty	
	*r*	*p*-Values	*r*	*p*-Values	*r*	*p*-Values
Age ^a^	0.389	< 0.001	-0.500	< 0.001	0.511	< 0.001
BMI ^a^	0.097	0.109	0.132	0.030	-0.220	< 0.001
SNAQ score^b^	-0.365	< 0.001	0.220	< 0.001	-0.252	< 0.001
HDMD score^b^	0.348	< 0.001	-0.310	< 0.001	0.364	< 0.001
HAMD score ^b^	0.306	< 0.001	-0.295	< 0.001	0.337	< 0.001
SSRS score^b^	-0.320	< 0.001	0.463	< 0.001	-0.503	< 0.001
MLHFQ score ^a^	0.370	< 0.001	-0.228	< 0.001	0.272	< 0.001

^a^ Values are analyzed by Pearson correlation analysis

^b^ Values are analyzed by Spearman correlation analysis

Abbreviations: BMI = body mass index; SSRS = Social Support Rating Scale; MLHFQ = Minnesota Living with Heart Failure Questionnaire; SNAQ = Simplified Nutrition Appetite Questionnaire; HAMD = the Hamilton Depression Scale; HAMA = the Hamilton Anxiety Scale

### Independent influencing factors of CF

The findings from the logistic regression analysis indicate that BMI, blood pressure level, nutrition status, and social support were all independent factors of CF (*p* < 0.05). Body mass index (*OR* = 0.826, 95%CI = 0.726–0.938), blood pressure level (*OR* = 2.323, 95%CI = 1.105–4.882), nutrition status (*OR* = 0.820, 95%CI = 0.671–0.979), and social support (*OR* = 0.745, 95%CI = 0.659–0.842) are independent influencing factors on CF. Table 3 displays the specific outcomes.

**Table 3:** Analysis of logistic regression models in CHF patients

Types of CF^a^	Variable	Beta	S.E.	*p*-value	Exp(B)	95% CI	
						Lower	Upper
R-CF	BMI	-0.052	0.059	0.373	0.949	0.845	1.065
	Combined with hypertension	0.333	0.354	0.348	1.395	0.696	2.794
	Combined with cerebral disease	-0.014	0.364	0.969	0.986	0.484	2.011
	SNAQ score	-0.019	0.092	0.838	0.981	0.819	1.176
	HAMD score	0.044	0.071	0.540	1.044	0.909	1.201
	HAMA score	0.031	0.075	0.678	1.032	0.890	1.195
	SSRS score	-0.193	0.060	**0.001**	0.824	0.733	0.927
	MLHFQ score	0.012	0.016	0.439	1.013	0.981	1.045
PR-CF	BMI	-0.192	0.065	**0.003**	0.826	0.726	0.938
	Combined with hypertension	0.843	0.379	**0.026**	2.323	1.105	4.882
	Combined with cerebral disease	-0.036	0.378	0.924	0.965	0.460	2.024
	SNAQ score	-0.210	0.096	**0.029**	0.810	0.671	0.979
	HAMD score	0.017	0.073	0.817	1.017	0.881	1.174
	HAMA score	0.090	0.078	0.246	1.094	0.940	1.274
	SSRS score	-0.294	0.062	**< 0.001**	0.745	0.659	0.842
	MLHFQ score	0.024	0.017	0.144	1.025	0.992	1.059

^a^ Taking none cognitive frailty as a reference

Abbreviations: S.E. = standard error; CF = cognitive frailty; R-CF = reversible cognitive frailty; PR-CF = potentially reversible cognitive frailty; BMI = body mass index; SNAQ = Simplified Nutrition Appetite Questionnaire; HAMD = the Hamilton Depression Scale; HAMA = the Hamilton Anxiety Scale; SSRS = Social Support Rating Scale; MLHFQ = Minnesota Living with Heart Failure Questionnaire

## Discussion

This study aimed to examine the prevalence of cognitive frailty (CF) in patients with chronic heart failure (CHF) and identify any modifiable risk factors. Our findings revealed a high prevalence of CF in this population, with BMI, blood pressure level, nutrition status and social support being identified as significant modifiable risk factors for CF.

In this study, it was found that the total prevalence of CF in patients with CHF was 49.4% [95%CI = 43.5–55.4%], with reversible cognitive frailty at 20.7% and potentially reversible cognitive frailty at 28.8%. Researchers from South Korea [29] and Japan [12] evaluated heart failure patients over the age of 65 and discovered that 34.5% and 23% of patients had CF, respectively. They did not investigate the two types of CF separately. However, Yao et al. investigated the prevalence of frailty and cognitive impairment in hospitalized patients with cardiovascular diseases and discovered that only 8% of patients had both conditions [30]. This difference may be due to the fact that Yao’s study included hospitalized patients with various cardiovascular disease, whereas our study only included CHF patients. Meanwhile, we used different assessment tools. Currently, cognitive and frailty assessment tools are commonly used to assess CF, but uniform assessment standards have yet to be developed. As the terminal stage of various cardiovascular diseases, CHF has a long course that involves many different systems and mechanisms. These include brain changes, vascular mechanisms, inflammation, hormones, sarcopenia, oxidative stress process, mitochondrial dysfunction and intestinal microbiome changes, all of which have a significant impact on physical frailty and cognitive function [11]. Simultaneously, it was found that the prevalence of CF in the elderly and middle-young patients undergoing maintenance hemodialysis was 35.8% and 8.8%, respectively, indicating that middle-young patients are equally susceptible to CF [19]. Our study found that the prevalence of CF was 17.1% among middle-young CHF patients, suggesting that the prevalence of CF in CHF patients of different age ranges should not be ignored. Previous studies on CF have focused on the elderly population, with little attention paid on middle-young demographic. Despite the limited number of middle-young patients included in this study, we found a significant prevalence of CF in this population. While CF has been extensively studied in the field of geriatrics, more research is needed to understand the current status of CF in middle-aged individuals. For middle-young CHF patients with CF, appropriate interventions should be implemented promptly to reverse CF, prevent its worsening with age, and reduce the risk of poor cardiovascular outcomes.

In this study, it was found that BMI was a modifiable risk factor of CF in patients with CHF (*OR* = 0.826, 95%CI = 0.726–0.938). Rietman et al. conducted a cohort research which discovered a slight linear correlation between BMI and CF, with higher BMI being associated with more severe CF [31]. However, other studies have found opposite results, with a higher BMI being linked to a lower risk of CF [32]. Therefore, the relationship between BMI and CF remains contentious. By reviewing data from the CLHLS project, Ju et al. discovered a U-shaped correlation between BMI, waist circumference (WC), and frailty in elderly Chinese women [33]. However, the biological mechanism underlying this relationship remains unknown. Further research is needed to identify additional indicators with predictive value. The study found that only being overweight was a risk factor for CF, while being underweight may also affect CF in patients with CHF. However, the specific effect and degree of influence of BMI require further investigation.

Nutrition status is another modifiable risk factor of CF (*OR* = 0.810, 95%CI = 0.671–0.979). Numerous studies have shown that malnutrition is a significant risk factor for CF [34], and it is negatively correlated with nutrition status. Malnutrition can lead to weight loss, muscle tissue loss, body wasting, and other physical frailties, ultimately contributing to the development of CF. Furthermore, the lack of essential nutrients such as serum proteins, vitamins, and trace elements can affect cognitive function, which is also a crucial factor in the development and progression of physical frailty and cognitive impairment [35]. Zupo et al. found that malnourished elderly people with CF had a higher mortality rate [36], highlighting the urgent need to improve the nutrition status of patients with CF. Consequently, we aim to substantiate the impact of nutrition status on CF in patients with CHF through forthcoming clinical trials.

Patients with hypertension had a 2.323 times higher likelihood of developing CF compared to those without hypertension (95%CI = 1.105–4.882). Wang et al. found that 9.8% of elderly hypertensive patients had CF [18], While up to 28% of hypertension and diabetes patients who underwent physical examination in community health service centers had CF [34]. These findings suggest that the prevalence of CF in elderly hypertensive patients in China should not be ignored. Scholars have identified that frequent morbidity, such as hypertension and heart disease, as influential factor for CF [37]. This may indicate that hypertension, heart disease and CF are interrelated and interconnected. As a major cause of cardiovascular disease, hypertension can damage brain capillaries, leading to cognitive impairment and accelerating the development of dementia. These risk factors for cognitive impairment are associated with the onset and deterioration of PF. Consequently, there is a necessity to initiate clinical trials to investigate the impact of hypertension on CF in patients with CHF.

We have found that social support is a modifiable risk factor for CF (*OR* = 0.745, 95%CI = 0.659–0.842). Studies have shown that low social support is an independent risk factor of CF in the elderly [38]. Apart from CHF, Wang et al. found that social support was also an independent factor impacting CF in hypertensive patients [18]. We speculate that social support may be a factor influencing CF in other diseases. Lack of social support may lead to various psychological problems. However, this study did not find evidence that depression, anxiety, or other mental states are independent factors influencing CF in CHF patients. Hou et al. found that depressive symptoms do not directly affect CF in older adults, but rather, feelings of loneliness are the link between the two [39]. Loneliness can reflect social support to some extent. However, Wang et al. found that psychological distress could regulate the relationship between social support and the prevalence of CF [17]. Therefore, the relationship between mental state and CF remains vague and requires further study.

The study did not find old age and low education level to be significant risk factors for CF, despite their correlation in the univariate analysis. This discrepancy may be attributed to the specific population and region included in the study. Although we transformed some continuous variables, we re-analyzed the data using the original continuous variables. Variables associated with CHF were treated as additional independent variables associated with CF risk. The prevalence of HFrEF was higher in the non-CF group (41%) compared to the CF groups (27% and 25%), while the prevalence of HFpEF was higher in the CF groups. This would suggest that CF is more prevalent in diastolic dysfunction rather than systolic dysfunction, but further studies are needed. Importantly, more than half of the Non-CF patients had CHF for ≤ 1 year, while the CF groups had a longer duration. This is significant in the regression analysis, where we seek to identify modifiable risk factors for CF. Additionally, the Non-CF group had a higher prevalence in the lower NYHA classes. In this study, we also found a strong correlation between CHF and CF, particularly through the indicators associated with CHF. As a result, it is crucial to monitor the condition of CHF patients and provide them with relief from CF while maintaining disease stability.

In this study, we broadened the scope of our research by including patients above the age of 18. This is because middle-aged and young people are also at risk of cognitive frailty, which has been previously overlooked in studies that mainly focused on the elderly. We also categorized patients into two groups: reversible cognitive frailty and potentially reversible cognitive frailty to better understand the status quo of CF in CHF patients. We identified modifiable risk factors that influence CF in patients with CHF. Nonetheless, clinical trials are imperative to ascertain whether these modifiable risk factors have the potential to reverse CF in CHF patients. This study adds to the current situation in domestic research on cognitive frailty in CHF patients above the age of 18.

Although this study has yielded valuable insights, there are still some limitations that need to be addressed. Firstly, it is a single-center study, and the majority of patients are from nearby towns and cities, which limits the generalizability of the findings. Future multi-center studies with large sample sizes are needed to overcome this limitation. Secondly, this study is constrained by its cross-sectional design, which impedes our ability to establish the predictive value of the aforementioned factors for CF. We urge and encourage researchers to delve further into the relationship among these factors. Future investigations could benefit from non-clinical population controls and longitudinal follow-up studies to provide a more comprehensive understanding of these associations. Thirdly, prevalence estimates obtained from convenience sampling are strongly subjected to sampling bias. In future studies, we intend to employ a more judicious and rational sampling method to enhance the robustness and generalizability of our findings. Finally, no unique influencing factors connected to CHF are discovered in this investigation, which may be related to the disease severity of the included patients. Additionally, the baseline data included in this study are incomplete, and there may be undiscovered influencing factors that require further investigation.

## Conclusion

There is a high prevalence of CF among patients with CHF in China. Therefore, healthcare providers need to conduct a comprehensive assessment of individual CHF patients to identify and prevent risks at an early stage. We suggest conducting clinical trials to test the effects of these modifiable risk factors. Our study adds to the research on CF in patients with CHF. These findings augment the existing knowledge on CF in CHF patients and offer valuable reference for medical staff to implement effective measures for improving the CF status in clinical practice.

## Data Availability

The datasets analyzed in the current study are available from the corresponding author upon reasonable request.

## Abbreviations

CF: cognitive frailty
CHF: chronic heart failure
MoCA: Montreal Cognitive Assessment
BMI: body mass index
AD: Alzheimer’s disease
NYHA: New York Heart Association
LVEF: left ventricular ejection fraction
PF: physical frailty
SSRS: Social Support Rating Scale
SNAQ: Simplified Nutrition Appetite Questionnaire
HAMD: the Hamilton Depression Scale
HAMA: the Hamilton Anxiety Scale
MLHFQ: Minnesota Living with Heart Failure Questionnaire
WC: waist circumference
